# Pure Open Ankle Dislocation Leading to Isolated Lateral Ligament Complex Tear: A Rare Case

**DOI:** 10.7759/cureus.52855

**Published:** 2024-01-24

**Authors:** Taha El Aissaoui, Aboubacar Lawan, Adnane Lachkar, Najib Abdeljaouad, Hicham Yacoubi

**Affiliations:** 1 Traumatology and Orthopedics, Mohammed VI University Hospital, Faculty of Medicine and Pharmacy, Mohammed First University, Oujda, MAR; 2 Orthopedics, Mohammed VI University Hospital, Faculty of Medicine and Pharmacy, Mohammed First University, Oujda, MAR; 3 Orthopedic Trauma, Mohammed VI University Hospital, Faculty of Medicine and Pharmacy, Mohammed First University, Oujda, MAR

**Keywords:** antibiotic, pure ankle dislocation, debridement, open dislocation, ankle and foot

## Abstract

Pure open ankle dislocation is a rare orthopedic emergency characterized by the absence of associated bony lesions, necessitating prompt and immediate management to prevent disastrous complications.

This article details a distinctive case of open pure ankle dislocation in an 18-year-old female following a motorcycle accident, resulting in a 6-cm wound and a dislocated left ankle with a concurrent tear of the anterior talofibular ligament and calcaneofibular ligament. Immediate reduction under sedation was performed, followed by intensive debridement and ligament repair in the operating room. Postoperatively, the patient received antibiotic coverage for five days and underwent immobilization for six weeks. At the 18-month follow-up, the patient exhibited a complete range of motion with no reported pain or instability.

This study contributes to the existing literature by presenting a successful treatment paradigm, providing valuable insights for practitioners encountering similar cases.

## Introduction

Open pure ankle dislocation is a rare injury, accounting for only 0.065% of all ankle injuries and 0.46% of all ankle dislocations [1]. The relative weakness of the bones in relation to the strength of the supporting ligaments can explain the rarity of this type of injury [2].

Pure ankle dislocation often occurs due to a high traumatic energy mechanism, which usually leads to the complete tear of the lateral/medial collateral ligaments and even the tibiofibular syndesmosis [3]. Approximately 35% of patients suffering from lateral ligament injury experience instability, and up to 44% of patients have instability one year after non-operative treatment [4]. Long-term studies on the outcome still need to be improved, and the treatment protocol needs to be better established [5,6].

We present a unique case of open pure ankle dislocation associated with the tear of the anterior talofibular ligament and the calcaneofibular ligament.

## Case presentation

**Figure 1:**
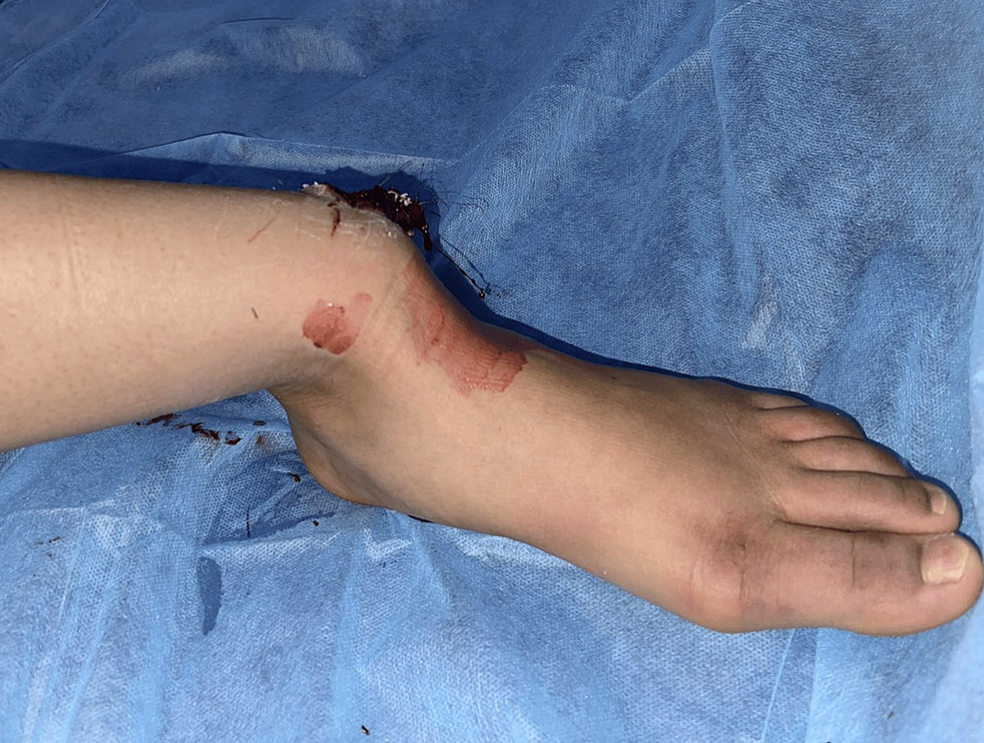
Clinical image of the left ankle deformation.

**Figure 2:**
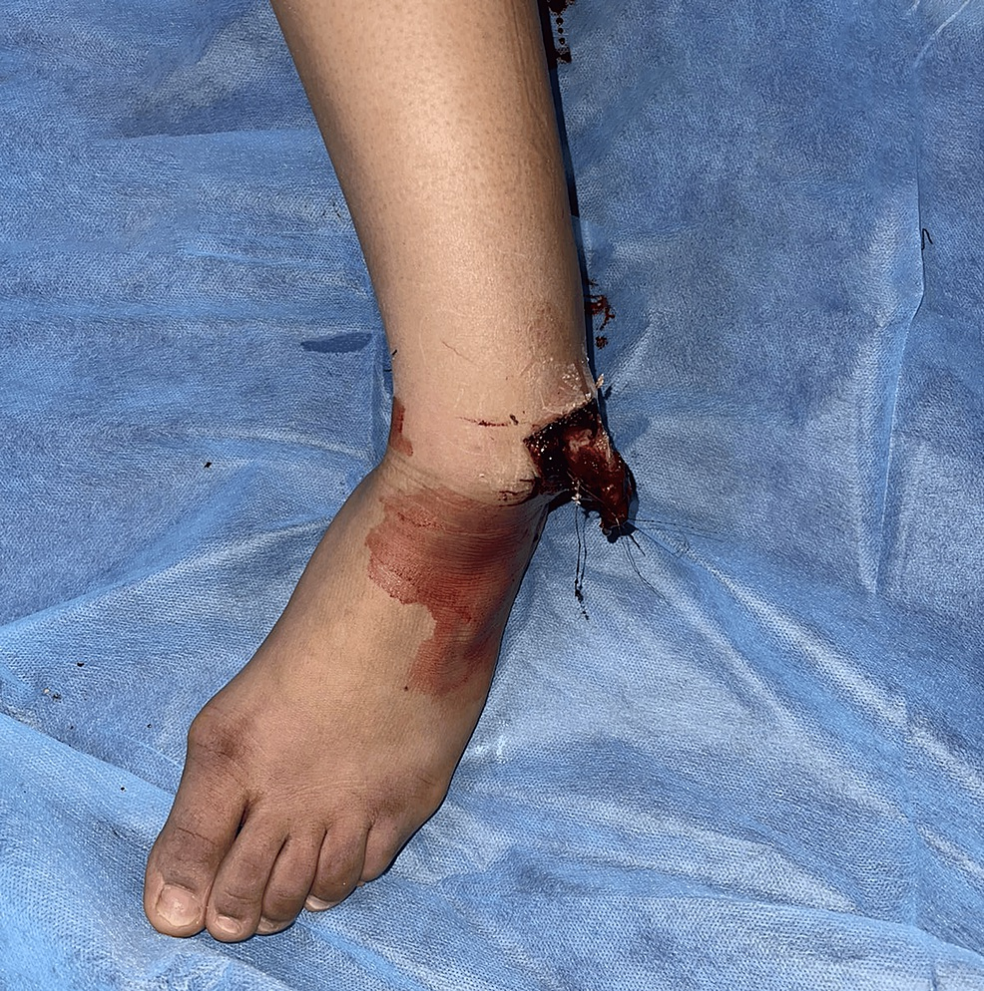
Clinical image of the left ankle deformation.

**Figure 3:**
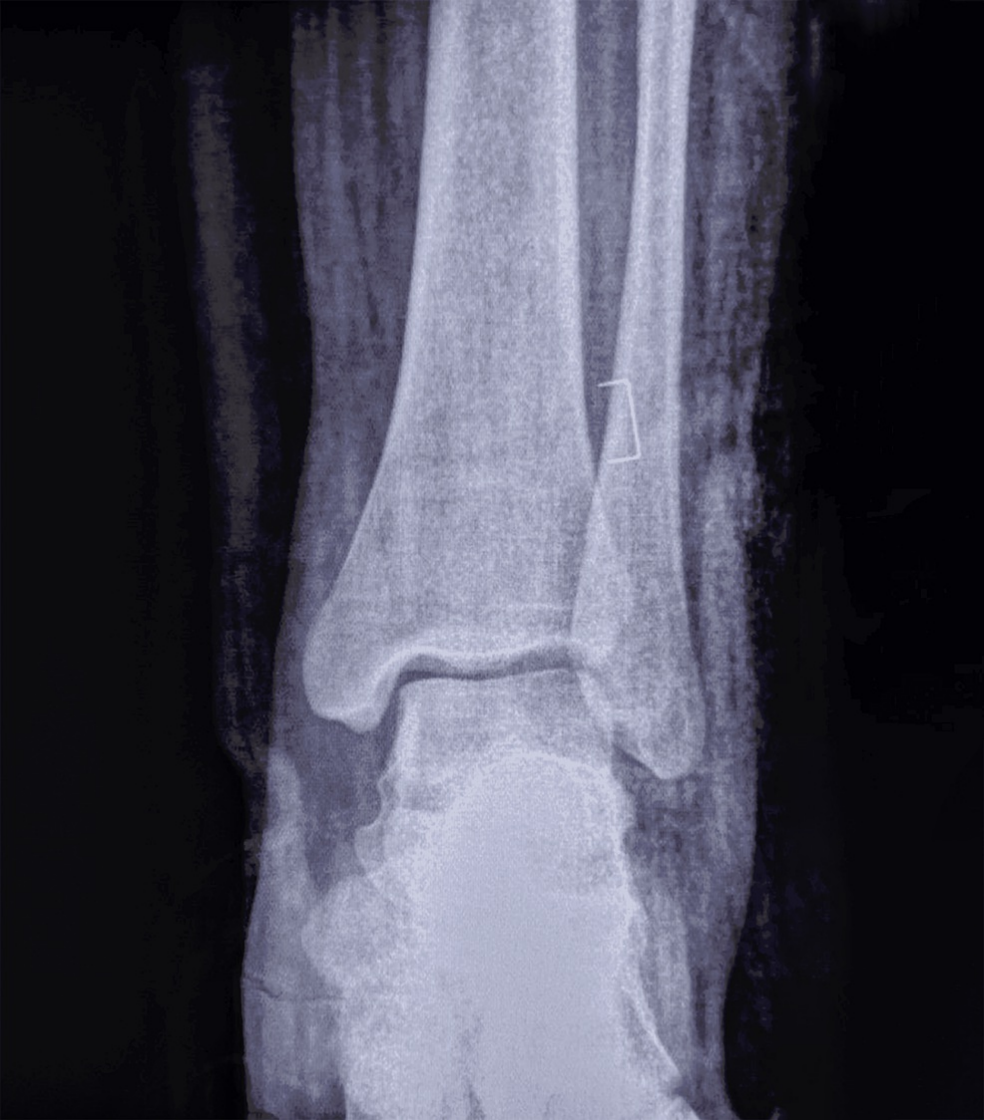
Anteroposterior post-reduction radiographic view of the left ankle.

**Figure 4:**
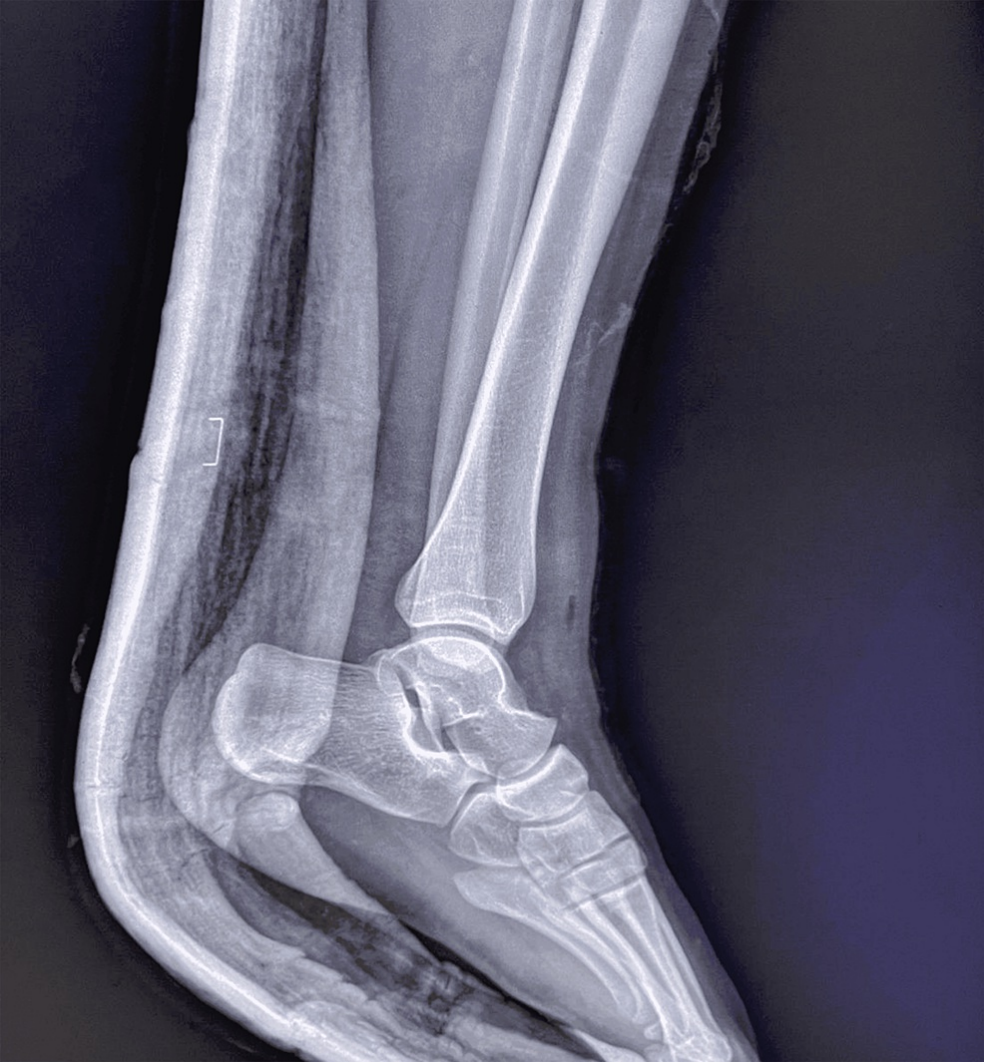
Lateral post-reduction radiographic view of the left ankle.

We present the case of an 18-year-old female with no medical history who arrived at our department 30 minutes after an accident involving a fall from a motorcycle. The patient presented with a deformed left ankle with a 6-cm wound on the lateral aspect (Figures 1, 2). Dorsalis pedis and posterior tibial pulses were not palpable during the physical evaluation, and the patient reported numbness. The dislocation was promptly reduced under sedation by applying longitudinal traction to the foot, as well as restoring pulses, normal capillary refill time, and neurological function. The patient received 1 g of amoxicillin/clavulanic acid, 160 mg of gentamicin, a tetanus vaccine, and tetanus antitoxin. Post-reduction radiographs revealed a well-reduced tibiotalar articulation with no evidence of bony lesions (Figures 3, 4).

**Figure 5:**
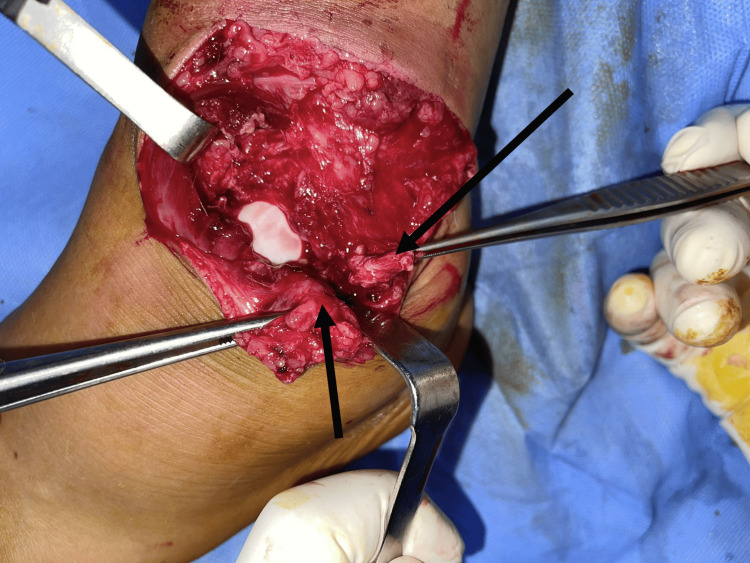
Intraoperative image of the left ankle illustrating a tear of the articular capsule and lateral ligament complex.

**Figure 6:**
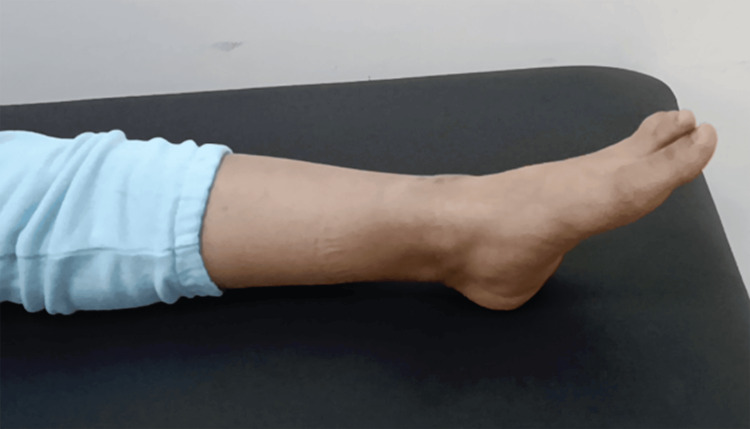
Clinical image demonstrating full flexion range of motion.

**Figure 7:**
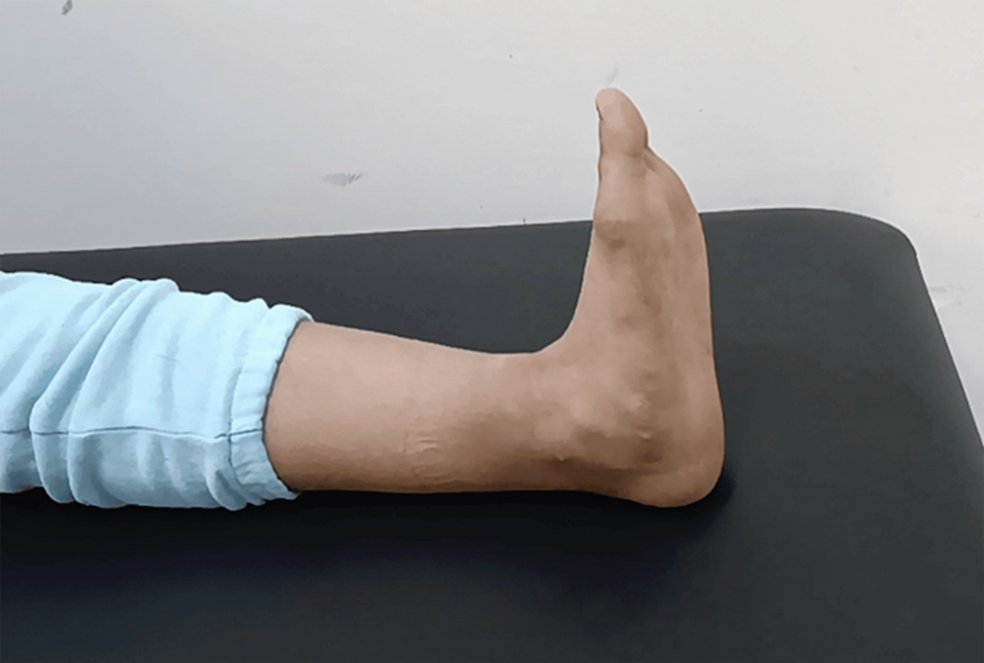
Clinical image illustrating full extension range of motion.

In the operating room, we performed intensive debridement, followed by a deeper exploration of the ankle joint, which revealed a tear of the anterior articular capsule, the anterior talofibular ligament, and the calcaneofibular ligament, associated with ankle lateral instability (Figure 5). No foreign bodies or osteochondral damage were found. We closed the capsule, repaired both ligaments by direct suture, and closed the wound. The patient received a five-day course of intravenous amoxicillin/clavulanic acid at a dose of 1 g three times per day, as well as gentamicin for two days at a dose of 160 mg per day. Additionally, the patient was immobilized with a posterior neutral splint for six weeks and received enoxaparin sodium at a dose of 4,000 IU per day during this period. The patient began progressive weight-bearing from the seventh week, along with an adapted rehabilitation program to restore proprioception, range of motion, and muscle strength. After 18 months of follow-up, the patient regained full range of motion with no pain or instability (Figures 6, 7).

## Discussion

Ankle pure dislocations are extremely rare [7], often occurring in male patients with an incidence of approximately 0.065% [1], and commonly associated with high-energy trauma, sports injuries, and traffic accidents [8]. According to Fahey and Murphy [9], these injuries may manifest in the following five presentations: anterior, posterior, medial, lateral, and combined. More than 50% of these dislocations are posteromedial [10].

Several conditions can predispose to this kind of dislocation, such as medial malleolus dysplasia, lack of talus coverage, ligamentous laxity, previous sprains, and weakness of the peroneal muscle [11]. In our case, the patient did not present any of these factors.

Neurovascular injuries are documented in 19% of cases before reduction [1]. As a result, researchers have a consensus to advocate for immediate reduction as soon as possible, emphasizing that imaging modalities should not cause any delays in the process [1,12]. Due to the absence of both dorsalis pedis and posterior tibial pulses, coupled with the noted numbness during the physical examination of our patient, we promptly proceeded with the dislocation reduction under sedation before conducting a radiographic assessment.

According to the literature, successful reduction requires the following steps: complete relaxation of the muscles by anesthesia or sedation and knee flexion to relax the pull of the sural triceps on the calcaneus [11,12], followed by longitudinal traction associated with manipulation in the opposite direction of the dislocation mechanism [11].

Ligament reconstruction remains a subject of controversial discussion [10]. Toohey and Worsing [13] found that ligamentous repair did not alter the outcome after surgical repair of the ligaments in two of six cases. Colville et al. [14] repaired the lateral ligaments in four of five open dislocations. The patient without repair developed late instability at five years, while the others had good long-term functional results. Ucar et al. [15] reported favorable long-term results without ligament or capsule repair in open pure ankle dislocations. In our view, acute repair spares the patient a second surgical intervention in case of instability. It ultimately ensures easier anatomical repair, considering the potential challenges posed by scar tissues and fibrosis in the case of late or deferred surgery.

The means of stabilization are varied. Bakshi [16] used two thick Kirshner wires to stabilize the ankle joint for six weeks, approximating the ligament remnant and joint capsule. The outcome showed excellent results. Bhullar et al. [17] primarily repaired only the lateral ankle ligament and immobilized the joint with a bulky Jones splint. Finally, Sayit et al. [18] simultaneously relied on an external fixator and ligament repair, obtaining a good functional outcome after one year. According to the literature, an average immobilization of six weeks is recommended [1,3,9].

Degenerative changes represent 25% of complications, with avascular necrosis, joint stiffness, and instability being rare [19]. Our patient has not presented any complications so far after a follow-up of two years.

## Conclusions

In conclusion, this report details a rare case of open pure ankle dislocation resulting in an isolated tear of the lateral ligament complex. Through a comprehensive exploration of the patient's presentation, surgical intervention, and follow-up, the study provides valuable insights into the intricacies of managing such uncommon injuries. The immediate reduction of the dislocation, coupled with acute repair, proves effective in achieving favorable long-term outcomes, avoiding complications, and preserving anatomical integrity. The diverse means of stabilization discussed underscore the importance of tailored approaches in addressing the complexities associated with this injury. This case report adds to the existing literature on ankle pure dislocations, emphasizing the significance of prompt intervention and presenting a successful treatment paradigm for practitioners to consider in similar cases.
